# Genomic Prediction of Average Daily Gain, Back-Fat Thickness, and Loin Muscle Depth Using Different Genomic Tools in Canadian Swine Populations

**DOI:** 10.3389/fgene.2021.665344

**Published:** 2021-06-03

**Authors:** Siavash Salek Ardestani, Mohsen Jafarikia, Mehdi Sargolzaei, Brian Sullivan, Younes Miar

**Affiliations:** ^1^Department of Animal Science and Aquaculture, Dalhousie University, Truro, NS, Canada; ^2^Canadian Centre for Swine Improvement, Ottawa, ON, Canada; ^3^Centre for Genetic Improvement of Livestock (CGIL), Department of Animal Biosciences, University of Guelph, Guelph, ON, Canada; ^4^Department of Pathobiology, University of Guelph, Guelph, ON, Canada; ^5^Select Sires Inc., Plain City, OH, United States

**Keywords:** BayesC, genomic prediction, GBLUP, single-step GBLUP, swine

## Abstract

Improvement of prediction accuracy of estimated breeding values (EBVs) can lead to increased profitability for swine breeding companies. This study was performed to compare the accuracy of different popular genomic prediction methods and traditional best linear unbiased prediction (BLUP) for future performance of back-fat thickness (BFT), average daily gain (ADG), and loin muscle depth (LMD) in Canadian Duroc, Landrace, and Yorkshire swine breeds. In this study, 17,019 pigs were genotyped using Illumina 60K and Affymetrix 50K panels. After quality control and imputation steps, a total of 41,304, 48,580, and 49,102 single-nucleotide polymorphisms remained for Duroc (*n* = 6,649), Landrace (*n* = 5,362), and Yorkshire (*n* = 5,008) breeds, respectively. The breeding values of animals in the validation groups (*n* = 392–774) were predicted before performance test using BLUP, BayesC, BayesCπ, genomic BLUP (GBLUP), and single-step GBLUP (ssGBLUP) methods. The prediction accuracies were obtained using the correlation between the predicted breeding values and their deregressed EBVs (dEBVs) after performance test. The genomic prediction methods showed higher prediction accuracies than traditional BLUP for all scenarios. Although the accuracies of genomic prediction methods were not significantly (*P* > 0.05) different, ssGBLUP was the most accurate method for Duroc-ADG, Duroc-LMD, Landrace-BFT, Landrace-ADG, and Yorkshire-BFT scenarios, and BayesCπ was the most accurate method for Duroc-BFT, Landrace-LMD, and Yorkshire-ADG scenarios. Furthermore, BayesCπ method was the least biased method for Duroc-LMD, Landrace-BFT, Landrace-ADG, Yorkshire-BFT, and Yorkshire-ADG scenarios. Our findings can be beneficial for accelerating the genetic progress of BFT, ADG, and LMD in Canadian swine populations by selecting more accurate and unbiased genomic prediction methods.

## Introduction

In the pork industry, genetics has a pivotal role in improving the economically important traits, such as meat quality, growth, and reproductive performance (Badke et al., 2014). The genetic improvement has been spectacularly successful using traditional genetic improvement tools, such as best linear unbiased prediction (BLUP) method (Henderson, 1975); however, the genetic gain achieved is relatively slow for traits that are expensive and difficult to measure such as those measured postmortem (Dekkers et al., 2010; Wolc et al., 2011; Miar et al., 2015). On the other hand, evaluation methods based on genotypic information or genomic evaluation are being used increasingly in modern pig breeding industry (Badke et al., 2014) because of their accelerative role in genetic improvement specifically through augmentation of predictive ability.

In genomic evaluation, the effects of markers are estimated based on genomic and phenotypic data in the reference group, and then it is used to predict genomic breeding values (GEBVs) in the validation group (Hayes and Goddard, 2001). The accuracy of genomic prediction can be influenced by several factors such as the reference population size (Samore and Fontanesi, 2016), genetic architecture of the trait, marker density, and method assumptions used for prediction (Zhang et al., 2019). Several former studies have compared different methods on genomic prediction accuracy in livestock species such as cattle (Luan et al., 2009; Guo et al., 2017; Mehrban et al., 2017; Li et al., 2018; Mrode et al., 2018) and mink (Karimi et al., 2019). However, there are still limited numbers of literature in pig (Song et al., 2017; Jafarikia et al., 2018; Zhang et al., 2018). Some popular genomic evaluation approaches such as genomic BLUP (GBLUP) and Bayesian methods have been widely used in the recent studies. For example, Zhang et al. (2018) showed higher predictive ability of BayesB method over GBLUP for average daily feed intake in a small population (*n* = 1,363) of Duroc pigs. The single-step GBLUP (ssGBLUP) method was considerably more accurate than GBLUP and BayesR for growth traits in a larger population (*n* = 2,084) of Yorkshire breed (Song et al., 2017). The differences between these approaches are their assumptions, for example, about the distribution of marker effects (Hayes and Goddard, 2001). Therefore, determining the most accurate method for genomic evaluation of different swine breeds is an important step of selective breeding.

Several genomic prediction models of GBLUP (Badke et al., 2014; Song et al., 2017, 2019b, 2020; Jafarikia et al., 2018; Zhang et al., 2018), ssGBLUP (Song et al., 2017, 2019b; Thekkoot et al., 2018; Hong et al., 2019; Lopez et al., 2019; Zhou et al., 2019; Aliakbari et al., 2020), and BayesC (Esfandyari et al., 2016; Song et al., 2020) have frequently been used for prediction of various traits in the previous studies in swine. The main assumption of GBLUP method is based on the infinitesimal model (i.e., the genetic variation of the trait was explained by a large number of loci) (Karaman et al., 2016, 2018) that has been widely used in genomic evaluation, principally due to its ease of implementation, in developed countries’ breeding programs, such as Holstein cattle breeding programs in Canada^1^. Similar to GBLUP method, the infinitesimal model is assumed in ssGBLUP method. The pivotal difference between ssGBLUP and GBLUP methods is applying a blended genetic relationship matrix (using genomic and pedigree relationship matrices) in the ssGBLUP method (Legarra et al., 2009). This method has been implemented in developing countries and breeding companies with small number of genotyped animals due to the improving role of blended relationship matrix in increasing the accuracy of predicted GEBVs (Mrode et al., 2018). This method can also allow detecting the conflicts of pedigree and regulating the relationship between genotyped and non-genotyped animals (Amaya Martínez et al., 2020). Additionally, breeding values for non-genotyped animals can be obtained simultaneously using ssGBLUP method, which is not the case in GBLUP analysis (Tsuruta et al., 2013; Misztal et al., 2014). The Bayesian genomic evaluation approaches (A,B,C, etc.) are nonlinear methods and are mostly applied using Markov chain Monte Carlo (MCMC) algorithm (Iheshiulor et al., 2017). Despite the linear genomic evaluation methods (e.g., ssGBLUP and GBLUP), some Bayesian methods (e.g., BayesCπ) assume that the genetic variation is explained by a fewer number of loci. This characteristic can be helpful to improve the evaluation accuracies for traits where their genetic architecture violates the infinitesimal model assumption (Hayes and Goddard, 2001; VanRaden et al., 2008). However, GBLUP can be considerably faster than Bayesian approaches in terms of computational speed (Song et al., 2019b). In BayesC model (Kizilkaya et al., 2010), a common variance for single-nucleotide polymorphisms (SNPs) with nonzero effects is assumed instead of a locus-specific variance. Although assuming that the probability of SNPs with nonzero effects (π) is known, it might be problematic for some traits in BayesC model. Habier et al. (2011) developed the BayesC model through hypothesizing unknown π that can be estimated, and therefore its prior distribution becomes uniform (0, 1) (Habier et al., 2011).

The prediction ability of genomic evaluation methods, which is considerably affected by their assumptions, is an important factor for genetic improvement in swine breeding companies. Therefore, the main goal of our study was to compare the prediction accuracies of traditional BLUP with different popular genomic evaluation methods including GBLUP, ssGBLUP, BayesC, and BayesCπ for average daily gain (ADG), back-fat thickness (BFT), and loin muscle depth (LMD) traits in Canadian swine populations.

## Materials and Methods

### Ethics Statement

The hogs used in this study were cared for according to the Canadian Council on Animal Care (Olfert et al., 1993) guidelines.

### Animals, Genotyping, Quality Control, and Imputation

In this study, we used genotypic and phenotypic data of BFT, ADG, and LMD in three swine breeds of Duroc, Landrace, and Yorkshire. The BFT and LMD were measured by ultrasonic machine (B mode), and ADG was calculated using Eq. 1:

(1) $$\text{ADG}=\frac{\mathrm{Final}\,\mathrm{weight}-\mathrm{birth}\,\mathrm{weight}}{\text{Days}}$$

These phenotypes were collected from 2010 to 2019 and adjusted to the weight of 120 kg (Table 1). The average numbers of phenotyped boars and gilts per litter for Duroc, Landrace, and Yorkshire breeds are reported in Supplementary Table 1. In this study, phenotypic and genotypic information was collected from distributed swine breeding companies across Canada, which participated in the Canadian Swine Improvement Program coordinated by the Canadian Centre for Swine Improvement^2^.

**TABLE 1:** Number of animals with phenotypes and genotypes in reference and validation groups for back-fat thickness at 120 kg (BFT), average daily gain from birth to 120 kg (ADG), and loin muscle depth at 120 kg (LMD) in three breeds of Duroc, Landrace, and Yorkshire (validation groups were phenotyped after January 2019).

Trait	Breed	No. of phenotypes			No. of genotypes					
			
					Reference			Validation		
		Boar	Gilt/sow	Total	Boar	Gilt/sow	Total	Boar	Gilt/sow	Total
BFT (mm)	Duroc	24,461	25,588	50,049	3,690	2,184	5,874	504	270	774
	Landrace	32,304	50,259	82,563	2,519	2,371	4,890	467	4	471
	Yorkshire	33,421	60,916	94,337	2,160	2,455	4,615	386	7	393
ADG (g/day)	Duroc	24,470	25,590	50,060	3,691	2,184	5,875	504	270	774
	Landrace	32,311	50,288	82,599	2,519	2,372	4,891	467	4	471
	Yorkshire	33,428	60,947	94,375	2,161	2,455	4,616	385	7	392
LMD (mm)	Duroc	24,461	25,588	50,049	3,690	2,184	5,874	504	270	774
	Landrace	32,304	50,259	82,563	2,519	2,371	4,890	467	4	471
	Yorkshire	33,421	60,916	94,337	2,160	2,455	4,615	386	7	393

Animals in the reference (*n* = 4,615–5,875) and validation (the performance tested animals after January 2019, *n* = 392–774) groups were genotyped with Illumina 60K (Illumina Inc., San Diego, CA, United States) or Affymetrix 50K (Affymetrix, Santa Clara, CA, United States) panels (Table 1). Quality control was performed by removing SNPs with minor allele frequency <0.05, call rate <0.9, and Hardy–Weinberg *P* < 0.0001. After quality control steps, the remaining SNPs (52,025 for all breeds) were used for imputation of the missing genotypes with FImpute 2.2 software (Sargolzaei et al., 2014). After the imputation step, 1,341 SNPs on sex chromosomes were discarded.

### Statistical and Genetic Analyses

#### Traditional Estimated Breeding Value

Model 1 was used for estimation of breeding values of each animal using the AIREMLF90 1.61 software (Misztal et al., 2002):

$$\mathrm{Model}\,1.y_{c}=1\,\mu +\mathrm{Xb}+\mathrm{Za}+\mathrm{Wu}+e,$$

where *y* is the vector of phenotypic data, μ is the overall mean, *X* is the incidence matrix relating fixed effects of herd–year–season–sex to phenotypes, *b* is the vector of fixed effects, *Z* is the incidence matrix relating phenotypes to additive genetic effects, *a* is the vector of additive genetic effects, *W* is the incidence matrix relating phenotypes to random common litter effects, *u* is the vector of random common litter effects, and *e* is the vector of random residual effects. It was assumed that $a∼N\,\left(0,A\,\sigma_{a}^{2}\right)$, $u∼N\,\left(0,I\,\sigma_{u}^{2}\right)$, and$e∼N\,\left(0,I\,\sigma_{e}^{2}\right)$, where *A* is the pedigree-based relationship matrix, $\sigma_{a}^{2}$ is the variance of additive genetic effects, $\sigma_{u}^{2}$ is the variance of common litter effects, *I* is the identity matrix, and $\sigma_{e}^{2}$ is the residual variance. The estimated variance components (before and after performance tests) using AIREMLF90 implemented in BLUP model (Model 1) are reported in Supplementary Table 2. The estimated breeding values (EBVs) of parents were used to calculate parent average EBV (PA) for each animal using the following equation:

(2) $$\text{PA}=\frac{\text{EBV}\,\left(\text{sire}\right)+\mathrm{EBV}\,\left(\mathrm{dam}\right)}{2}.$$

#### GBLUP

After calculating the deregressed EBVs (dEBVs) as pseudophenotypes according to the approach proposed by Garrick et al. (2009), the GBLUP method was performed using Model 2 implemented in SNP1101 software 1.0 (Sargolzaei, 2014).

$$M\,o\,d\,e\,l\,2.y_{c}=1\,\mu +Z\,g+e$$

In Model 2, *y_C_* is the vector of dEBVs (reference population) as pseudophenotypes, μ is the overall mean effect, *Z* is the incidence matrix relating phenotypes (dEBVs) to GEBVs, *g* is the vector of GEBVs, and *e* is the vector of random residual effects. It was assumed that $g∼N\,\left(0,G\,\sigma_{g}^{2}\right)$ and $e∼N\,\left(0,W\,\sigma_{e}^{2}\right)$, where *G* is the genomic relationship matrix, $\sigma_{g}^{2}$ is the genomic variance, *W* is a diagonal matrix of residual weights, and $\sigma_{e}^{2}$ is the residual variance. The residual weights ($w_{i}=\frac{1-r_{i}^{2}}{r_{i}^{2}})$ were calculated based on the reliability of dEBVs ($r_{i}^{2}$) as described by Garrick et al. (2009).

The genomic relationship matrix was constructed as VanRaden (2008) described:

(3) $$G=\frac{Z\,Z^{\prime }}{2\,\sum_{j=1}^{i}p_{j}\,\left(1-p_{j}\right)},$$

where *Z* is the allele frequency adjusted genotype matrix with 0–2*p_j_*(for AA genotype), 1–2*p_j_* (for AB genotype), and 2–2*p_j_* (for BB genotype) elements, and dimension of the number of individuals by the number of markers. *p_j_* is the minor allele frequency for *j*-th marker.

The estimated variance components obtained from the “*aireml*” procedure (in SNP1101 software) implemented in GBLUP model (Model 2) are reported in Supplementary Table 2. The genomic relationship matrix visualization was performed using a custom-made script in python.

#### Single-Step GBLUP

The ssGBLUP analysis (Legarra et al., 2009; Christensen and Lund, 2010) was performed using AIREMLF90 1.61 software (Misztal et al., 2014). Model 3 was used for single-step genomic evaluation of each animal:

Model⁢3.y=1⁢μ+Xb+Zg+Wu+e

where *y*, μ, *X*, *b*, *W*, *u*, and *e* were explained in Model 1, *Z* is the incidence matrix relating phenotypes to GEBVs, and *g* is the vector of GEBVs. It was assumed that the variance of genomic effects ($\sigma_{g}^{2}$), variance of common litter effects ($\sigma_{u}^{2}$), and residual variance ($\sigma_{e}^{2}$) are governed by the normal distribution ($g∼N\,\left(0,H\,\sigma_{g}^{2}\right)$, $u∼N\,\left(0,I\,\sigma_{u}^{2}\right)$, and$e∼N\,\left(0,I\,\sigma_{e}^{2}\right)$, respectively). In ssGBLUP model, the *H* matrix was used, which was a combination of relationship matrices based on marker genotypes (*G*) and pedigree information (*A*). In the inverse of *H* matrix, *A*_*22*_ is the pedigree-based relationship matrix for genotyped animals, and τ and ω are the scaling factors, which both were set equal to one as the default option in AIREMLF90 1.61 software (Misztal et al., 2014). The blending factors of *G* (α) and *A* (β) matrices in the inverse of *H* matrix were set equal to 0.95 and 0.05, respectively, which were defined as $H^{-1}=A^{-1}+\left[\begin{array}{cc} 0 & 0\\ 0 & \tau \,{\left(\alpha \,G+\beta \,A_{22}\right)}^{-1}-\omega \,A_{22}^{-1} \end{array}\right]$ to avoid singularity problems and improve predictions (VanRaden, 2008; Lourenco et al., 2014). The estimated variance components from AIREMLF90 implemented in ssGBLUP model (Model 3) are reported in Supplementary Table 2.

#### Bayesian Approaches

In BayesC framework, the genomic evaluation was performed by implementing Model 4 and MCMC process in GS3 2.0 software (Legarra et al., 2011a) with the following criteria: NITER (number of iterations) = 100,000, BURNIN (beginning of the MCMC run) = 20,000, and THIN (thin interval) = 100. The applied prior of variance components were similar to the estimated variance components in BLUP model before performance test (Supplementary Table 2).

$$M\,o\,d\,e\,l\,4.y_{C}=1\,\mu \,\sum_{i=1}^{n}Z_{i}\,\alpha_{i}\,\delta_{i}+e$$

In Model 4, *y_C_* is the vector of pseudophenotypes as defined in Model 2, μ is the overall mean, *n* is the number of SNPs, *Z_i_* is the vector of genotype covariates, α_*i*_ is the allele substitution effect for SNP*_*i*_*, δ_*i*_ is an indicator for whether the SNP*_*i*_* has effect (1) or not (0), and *e* is the vector of random residual effects. The residuals were weighed based on the reliabilities of dEBVs as defined by Legarra et al. in GS3 software (Legarra et al., 2011a). For BayesCπ, the π prior as the probability level of an SNP having no effect was set equal to 0.99.

### Validation and Model Comparison

The accuracies of genomic predictions and PAs were calculated as the correlation between breeding values (GEBVs or PAs) of the validation group and their dEBVs after performance test. The standard errors of prediction accuracies were calculated using Eq. 4:

(4) $$\mathrm{Standard}\,\mathrm{error}=\frac{1-{\text{accuracy}}^{2}}{\sqrt{\mathrm{number}\,\mathrm{of}\,\mathrm{individuals}-1}}$$

**FIGURE 1:**
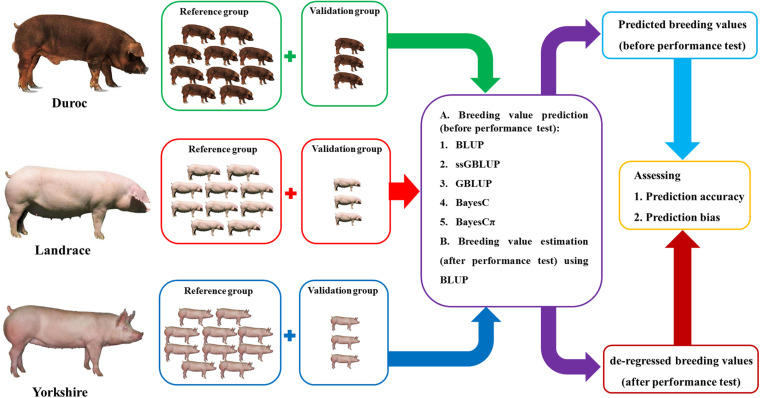
The summary of breeding value prediction workflow using the best linear unbiased prediction (BLUP), genomic BLUP (GBLUP), single-step genomic BLUP (ssGBLUP), BayesC, and BayesCπ methods in this study.

The regression coefficients of dEBVs (after performance test in January 2019) on predicted breeding values (before performance test in January 2019) were calculated to evaluate the bias of predictions (Figure 1). The regression coefficients and their standard errors were calculated using “*lm*” and “*summary*” functions in R 4.0.2 software (R Core Team, 2013).

The accuracy improvements were calculated using Eq. 5:

(5) $$\text{Im}\,\mathrm{provement}\,\mathrm{of}\,\mathrm{accuracy}=\left(\frac{\text{accuracy}\,\text{of}\,\text{GEBV}-\mathrm{accuracy}\,\text{of}\,\text{PA}}{\mathrm{accuracy}\,\mathrm{of}\,\mathrm{PA}}\right)\times 100.$$

Two criteria of accuracy and regression coefficient of dEBV on GEBV were used to compare the predictive ability of different genomic prediction methods. Additionally, a fixed model equation (Model 5) was employed to detect the significant differences of the prediction accuracies obtained from different methods.

$$\text{M}\,o\,d\,e\,l\,5.y_{i\,j}=\mu +M_{i}+B_{j}+e_{i\,j},$$

where *y* is the accuracy of prediction for the trait, μ is the overall mean, *M* is the fixed effect of prediction method *i*, *B* is the fixed effect of breed *j*, and *e* is the random residual effect for *i*-th method and *j*-th breed. The computational times of different scenarios with the same number of threads (*n* = 80) and memories (202 GB) were calculated using the default option of Slurm 20.02.3 software^3^ in Compute Canada server (Niagara cluster^4^). In this study, the computing time included the construction of genomic relationship matrix and its inverse, variance components estimation, and solving models.

## Results and Discussion

### Descriptive Statistics

The standard deviation, minimum and maximum values, and the coefficient of variation for each trait and breed are given in Table 2. The minimum (*n* = 50,060) and maximum (*n* = 94,375) numbers of phenotyped animals were observed for Duroc-ADG and Yorkshrie-ADG scenarios. The Landrace-BFT (14.27 mm), Duroc-ADG (736.72 g/day), and Duroc-LMD (72.61 mm) had the highest averages. The coefficients of variation ranged from 18.78 (Duroc) to 23.07 (Landrace), from 8.34 (Landrace) to 8.65 (Yorkshire), and from 8.25 (Duroc) to 8.83 (Yorkshire) for BFT, ADG, and LMD, respectively.

**TABLE 2:** Descriptive statistics of back-fat thickness at 120 kg (BFT), average daily gain from birth to 120 kg (ADG), and loin muscle depth at 120 kg (LMD) for Yorkshire, Landrace, and Duroc breeds.

Breed	Trait	n	Mean	SD	Min	Max	CV (%)
BFT (mm)	Duroc	50,049	12.5	2.55	4.9	32.8	18.78
	Landrace	82,563	14.27	3.29	5.1	39.4	23.07
	Yorkshire	94,337	14.26	3.04	5.8	39.2	21.30
ADG (g/day)	Duroc	50,060	736.72	62.56	354.4	1,072.1	8.49
	Landrace	82,599	711.74	59.34	396	1,077.5	8.34
	Yorkshire	94,375	706.97	61.16	353.9	1,061.6	8.65
LMD (mm)	Duroc	50,049	72.61	5.99	41.3	101.1	8.25
	Landrace	82,563	68.39	5.95	38.7	96.4	8.71
	Yorkshire	94,337	69.51	6.14	39.6	103.7	8.83

*n, number of animals per trait; SD, standard deviation; Min, minimum value; Max, maximum value; CV, coefficient of variation.*

### Validation of Genomic Evaluations

After quality control, removing SNPs on sex chromosomes, and imputation, the total numbers of 41,304, 48,580, and 49,102 SNPs remained for genomic evaluation of Duroc, Landrace, and Yorkshire breeds, respectively. Finding an accurate and unbiased genomic prediction method can be a lucrative strategy for genetic improvement of key traits in livestock species (Mrode et al., 2018). The predictive ability of genomic methods depends on various factors such as method hypothesis (Momen et al., 2018). However, limited numbers of literature are available in swine (Esfandyari et al., 2016; Song et al., 2017, 2019a; Zhang et al., 2018). Several genomic evaluation studies in livestock species such as cattle (Cardoso et al., 2014; Neves et al., 2014; Brown et al., 2016; Júnior et al., 2016; Silva et al., 2016; Costa et al., 2019) investigated and compared different genomic prediction methods; for example, Silva et al. (2016) showed the superior prediction accuracy of ssGBLUP over BayesCπ and GBLUP methods for residual feed intake (RFI) and feed conversion ratio (FCR) in Nelore cattle. Therefore, comparing different genomic prediction methods is important to detect more accurate methods for genomic evaluation of key traits in the swine breeding industry.

The average computational times for GBLUP, ssGBLUP, BayesC, and BayesCπ were 00:00:33, 00:18:47, 15:16:37, and 15:40:02 h, respectively. The computational times of GBLUP, ssGBLUP, BayesC, and BayesCπ ranged from 00:00:27 h (Yorkshire-BFT and Landrace-BFT) to 00:00:43 h (Duroc-LMD), 00:09:54 h (Duroc-LMD) to 00:31:43 h (Landrace-BFT), 14:21:19 h (Yorkshire-ADG) to 16:21:31 h (Duroc-ADG), and 14:16:28 h (Yorkshire-BFT) to 16:22:40 h (Duroc-LMD), respectively (Supplementary Table 3). The correlation between dEBVs (after performance test) and predicted GEBVs (before performance test) was considered as the accuracy of genomic prediction (Table 3). Moreover, the correlation between PAs (before performance test) and dEBVs (after performance test) was calculated as the accuracy of PAs (Table 3).

**TABLE 3:** The prediction accuracies (%), their standard errors and their improvement (%) over parent average EBV (PA) for back-fat thickness (BFT), average daily gain (ADG), and loin muscle depth (LMD) traits in Duroc, Landrace, and Yorkshire breeds.

Trait	Breed	PA	GBLUP		ssGBLUP		BayesC		BayesCπ	
						
		Accuracy	Accuracy	Improvement	Accuracy	Improvement	Accuracy	Improvement	Accuracy	Improvement
**BFT**	**Duroc**	13.9(3.5)	39.1(3.1)	181.4	39.2(3)	182.1	35.0(3.2)	151.4	42.4(3)	204.6
	**Landrace**	28.5(4.2)	49.2(3.5)	72.4(3.5)	52.7(3.3)	84.6	52.6(3.3)	84.4	50.8(3.4)	78.1
	**Yorkshire**	30.4(4.6)	42.4(4.1)	39.8(4.1)	44.7(4)	47.3	41.1(4.2)	35.6	41.2(4.2)	35.6
**ADG**	**Duroc**	5.7(3.6)	20.9(3.4)	266.4	23.7(3.4)	314.3	21.5(3.4)	276.7	19.3(3.5)	238
	**Landrace**	16.4(4.5)	33.0(4.1)	100.8	34.5(4.1)	110.0	26.6(4.3)	62.1	32.8(4.1)	99.7
	**Yorkshire**	12.0(5)	28.8(4.6)	140.7	26.2(4.6)	119.5	27.5(4.7)	129.8	29.5(4.6)	147
**LMD**	**Duroc**	3.7(3.6)	12.0(3.6)	225.6	12.6(3.5)	240.8	11.2(3.6)	202.6	10.3(3.6)	178.4
	**Landrace**	17.2(4.5)	22.5(4.4)	31.2	22.8(4.4)	33.1	18.3(4.5)	6.4	25.1(4.3)	46.2
	**Yorkshire**	5.6(5)	21.6(4.8)	284.5	21.3(4.8)	277.9	17.7(4.9)	213.6	20.7(4.8)	267.4

*GBLUP, genomic BLUP; ssGBLUP, single-step genomic BLUP.*

The accuracies ranged from 13.9% (Duroc-PA) to 52.7% (Landrace-ssGBLUP) for BFT, from 5.7% (Duroc-PA) to 34.5% (Landrace-ssGBLUP) for ADG, and from 3.7% (Duroc-PA) to 25.1% (Landrace-BayesCπ) for LMD. The accuracy improvements over PA ranged from 35.6% (Yorkshire-BayesC) to 204.6% (Duroc-BayesCπ) for BFT, from 62.1% (Landrace-BayesC) to 314.3% (Duroc-ssGBLUP) for ADG, and from 6.4% (Landrace-BayesC) to 284.5% (Yorkshire-GBLUP) for LMD (Table 3). The prediction accuracies of genomic methods were not significantly (*P* > 0.05) different from each other based on Tukey test implemented in Model 5 (Supplementary Table 4). However, the prediction accuracies obtained from PA were significantly (*P* < 0.05) lower than those obtained from genomic methods except for BayesC in LMD trait (Supplementary Table 4). Moreover, the prediction accuracies obtained from genomic methods were significantly (*P* < 0.05) different among breeds except for Yorkshire-Duroc (for BFT) and Yorkshire-Landrace (for ADG and LMD).

### Back-Fat Thickness

**FIGURE 2:**
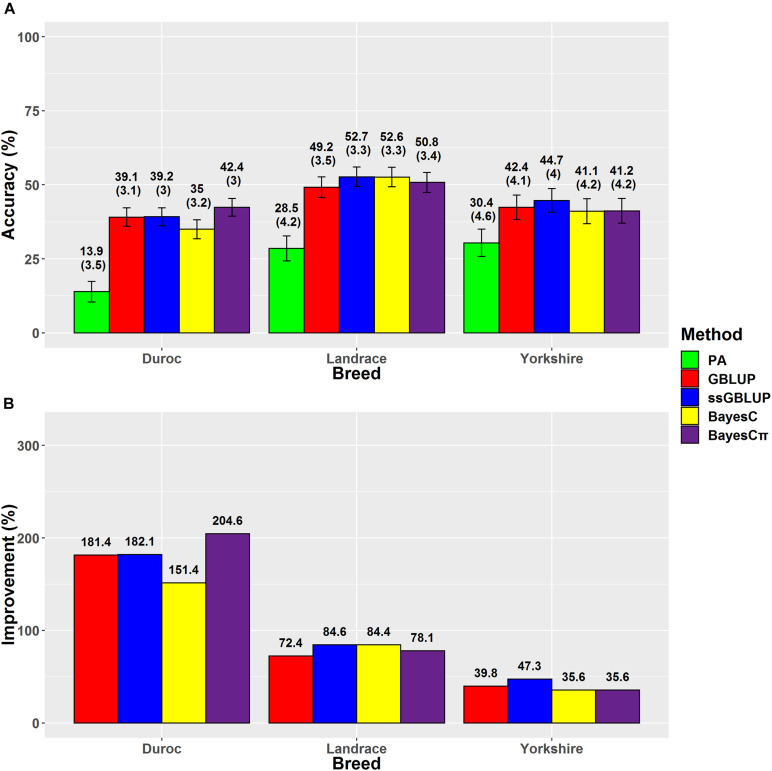
**(A)** The accuracies with their standard errors and **(B)** accuracy improvements obtained from genomic BLUP (GBLUP), single-step genomic BLUP (ssGBLUP), BayesC, BayesCπ, and parent average EBV (PA) methods for back-fat thickness.

The results demonstrated that ssGBLUP was the most accurate method for genomic evaluation of BFT in Landrace (52.7%) and Yorkshire (44.7%) breeds (Figure 2). The ssGBLUP method had the same prediction accuracy (44.1%) for BFT in Chinese Yorkshire population as our prediction (Zhou et al., 2019). However, the accuracy of ssGBLUP method for Yorkshire-BFT scenario was considerably higher than Thekkoot et al.’s (2018) result (30%), which might be due to their smaller number of genotyped animals in the reference group (*n* = 2,489). Higher prediction accuracies obtained from ssGBLUP for BFT in Yorkshire and Landrace breeds compared to Duroc breed might be due to the similarity of these breeds as maternal lines as well as using the larger population size (pedigree and phenotype data) of Yorkshire-BFT (*n* = 94,337) and Landrace-BFT (*n* = 82,563) in comparison with Duroc-BFT (*n* = 50,049). For BFT, the prediction accuracy superiority of ssGBLUP method over the other genomic prediction methods such as GBLUP was confirmed by previous studies in American Yorkshire breed (Song et al., 2017, 2019a). The regression coefficients of dEBVs on predicted GEBVs were estimated and applied as indices of prediction bias of the genomic evaluation methods (Table 4).

**TABLE 4:** Regression coefficient and their standard errors of deregressed EBV (dEBV) on predicted breeding values obtained from genomic BLUP (GBLUP), single-step genomic BLUP (ssGBLUP), BayesC, BayesCπ, and parent average EBV (PA) for back-fat thickness (BFT), average daily gain (ADG), and loin muscle depth (LMD) in Duroc, Landrace, and Yorkshire breeds.

Trait	Breed	PA	GBLUP	ssGBLUP	BayesC	BayesCπ
BFT	Duroc	0.90 (0.23)	1.11 (0.09)	1.60 (0.13)	0.58 (0.05)	1.12 (0.08)
	Landrace	0.76 (0.11)	1.15 (0.09)	1.14 (0.08)	0.62 (0.04)	1.05 (0.08)
	Yorkshire	1.16 (0.18)	1.22 (0.13)	1.31 (0.13)	0.55 (0.06)	1.11 (0.12)
ADG	Duroc	0.98 (0.62)	1.15 (0.19)	2.36 (0.34)	1.23 (0.20)	1.27 (0.23)
	Landrace	1.00 (0.28)	1.17 (0.15)	1.27 (0.16)	0.37 (0.06)	1.0 (0.13)
	Yorkshire	0.85 (0.36)	1.30 (0.22)	1.23 (0.23)	0.46 (0.80)	1.05 (0.17)
LMD	Duroc	0.58 (0.56)	1.21 (0.36)	1.48 (0.42)	0.40 (0.12)	0.83 (0.20)
	Landrace	0.75 (0.20)	1.01 (0.20)	0.90 (0.17)	0.32 (0.08)	0.90 (0.16)
	Yorkshire	0.34 (0.31)	1.15 (0.26)	0.97 (0.22)	0.27 (0.07)	0.82 (0.19)

The regression coefficients higher and lower than 1 indicate overestimation and underestimation, respectively. The ssGBLUP method showed higher prediction accuracies in Landrace-BFT and Yorkshire-BFT scenarios, but BayesCπ was the least biased method in these scenarios (1.06 for Landrace-BFT and 1.12 for Yorkshire-BFT). In Duroc-BFT scenario, BayesCπ (42.4%) and GBLUP (1.11) were the most accurate and least biased methods, respectively. The higher prediction accuracy of BayesCπ for Duroc-BFT scenario might be due to a low number of major-effect SNPs underlying genetic variation of BFT in Duroc breed (Zhang et al., 2020). However, Tukey test showed that the genomic prediction accuracies for BFT were not significantly (*P* > 0.05) different across breeds. In a previous genomic prediction study on a small population size of Canadian Duroc breed (*n* = 1,363), Zhang et al. (2018) showed the superiority of BayesRC approach for BFT. The BayesRC is a new method based on BayesR that combines prior biological datasets through explaining variant classes presumably to be enriched for causal polymorphisms (MacLeod et al., 2016). Compared to our results, they (Zhang et al., 2018) showed higher prediction accuracy (62 vs. 42.4%) for Duroc-BFT using whole-genome sequence data that might be due to using sequence data and a different method in their prediction.

### Average Daily Gain

**FIGURE 3:**
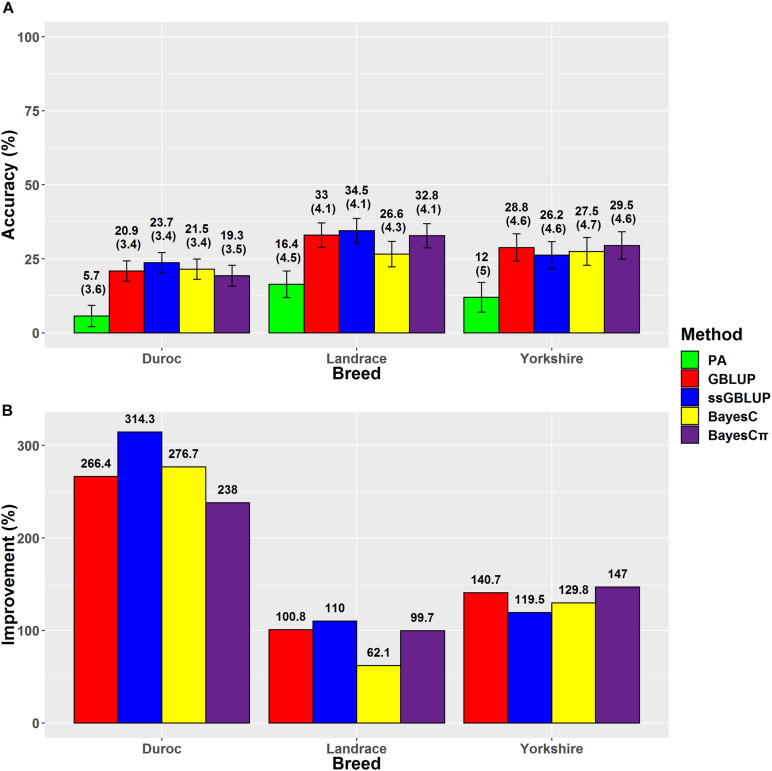
**(A)** The accuracies with their standard errors and **(B)** the accuracy improvements obtained from genomic BLUP (GBLUP), single-step genomic BLUP (ssGBLUP), BayesC, BayesCπ, and parent average EBV (PA) methods for average daily gain.

Our results indicated that ssGBLUP was the most accurate method (34.5%) for prediction of ADG in Landrace breed (Figure 3); however, BayesCπ was the least biased (1) method for ADG genomic evaluation (Table 4). Our prediction accuracy of ssGBLUP for Landrace-ADG (34.5%) was slightly higher than the results of Hong et al. (2019) (31–33%), who used different blending strategies of pedigree and genomic relationship matrices in ssGBLUP method. For Yorkshire-ADG, they obtained 21–22% accuracy of prediction using ssGBLUP method (Hong et al., 2019). In this study, BayesCπ was the most accurate (29.5%), and least biased (1.06) method for Yorkshire-ADG scenario. The prediction accuracy obtained from ssGBLUP method in Duroc-ADG scenario (23.7%) was similar to the study by Jiao et al. (2014) (24.10%) and slightly lower than that by Zhang et al. (2018) (25%). Jiao et al. (2014) used the Bayes A model in a small population size (*n* = 1,022) of Duroc breed in the reference group. The implementation of genomic multitrait models using ADG, FCR, and RFI traits might be beneficial for improvement of prediction accuracy for ADG in Duroc breed through implementing information from genetically correlated traits (Guo et al., 2014). Additionally, the prediction accuracy is considerably related to the genomic heritability, which is affected by the number of markers (Goddard, 2009). By comparing the genomic prediction abilities using different SNP densities (whole-genome sequence, 650K and 80K SNP panels) and prediction methods, Zhang et al. (2018) revealed that the density of SNPs could affect the prediction accuracy in a small population size of Canadian Duroc breed (*n* = 1,363) (Zhang et al., 2018). Although, except for BayesRC (25%) method, the GBLUP (12%), and BayesB (12%) methods showed lower prediction accuracies using the whole-genome sequence data in the study by Zhang et al. (2018) compared to our result (23.7%) for Duroc-ADG-ssGBLUP, these results can confirm the importance of population size, method assumptions, and SNP densities in genomic prediction analysis.

### Loin Muscle Depth

**FIGURE 4:**
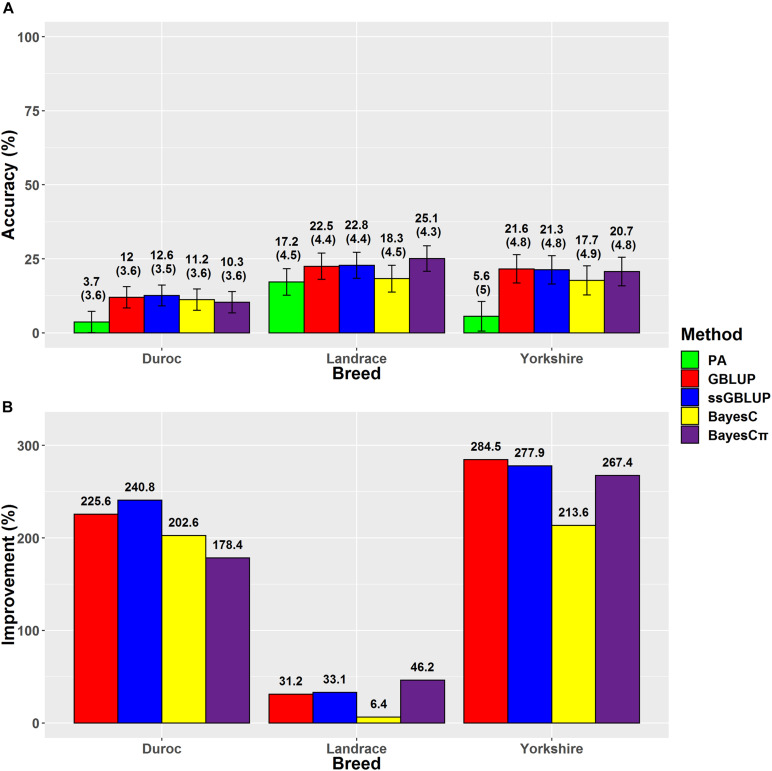
**(A)** The accuracies with their standard errors and **(B)** the accuracy improvements obtained from genomic BLUP (GBLUP), single-step genomic BLUP (ssGBLUP), BayesC, BayesCπ, and parent average EBV (PA) methods for loin muscle depth.

The highest accuracies for LMD in Duroc, Landrace, and Yorkshire breeds were derived from ssGBLUP (12.6%), BayesCπ (25.1%), and GBLUP (21.6%) methods, respectively (Figure 4). However, the regression coefficients followed a different trend for LMD scenarios. For LMD scenarios, the least biased methods were BayesCπ in Duroc (0.83), GBLUP in Landrace 1.01, and ssGBLUP (0.97) in Yorkshire breed. In this study, the highest genomic prediction accuracies for Yorkshire-LMD (21.6%) and Landrace-LMD (25.1%) scenarios were lower than the results by Jafarikia et al. (2018) for Yorkshire (38%) and Landrace (38%) that might be due to their larger reference group size of Canadian Yorkshire (*n* = 8,756) and Landrace (*n* = 6,754) pigs (Jafarikia et al., 2018), although, our genomic prediction accuracy (25.1%) for Landrace-LMD using the BayesCπ method was higher than the result of another study on Landrace (17.9–18.8%) pigs using different genomic relationship matrices (Sevillano et al., 2017). In Duroc-LMD scenario (GBLUP = 12%, ssGBLUP = 12.6%, BayesC = 11.2, and BayesCπ = 10.3%), the genomic prediction accuracies were lower than Landrace-LMD (GBLUP = 22.5%, ssGBLUP = 22.8%, BayesC = 18.3%, and BayesCπ = 25.1%) and Yorkshire-LMD (GBLUP = 21.6%, ssGBLUP = 21.3%, BayesC = 17.7%, and BayesCπ = 20.7%) that were similar to most of the scenarios for ADG and BFT traits. A small portion of these lower accuracies in Duroc scenarios might be due to slightly lower genomic relationships between validation and reference groups in Duroc (Supplementary Figure 1) compared to Landrace (Supplementary Figure 2) and Yorkshire (Supplementary Figure 3). Our results also showed the same patterns for genomic relationships among animals within the validation sets. However, the patterns were reverse in the pedigree-based relationships. The negative effects of weak genomic relationships between validation and reference groups on predictive ability were reported previously (Hayes et al., 2009a; Song et al., 2017).

The prediction accuracy is also affected by response variables through correlating predicted breeding values and response variables (adjusted phenotype, EBV, or dEBV) after performance test. The reliability of dEBV as a response variable is highly dependent on the population size of phenotyped animals (Mrode et al., 2018). In Duroc, the main reason for these low accuracies can be referred to the low reliabilities of dEBVs in the validation group derived from the smaller population size of phenotyped Duroc (50,049–50,060) compared to Yorkshire (94,337–94,375) and Landrace (82,563–82,599) (Table 1), whereas the reference population size of Duroc (5,874–5,875) was higher than Yorkshire (4,615–4,616) and Landrace (4,890–4,891). Additionally, the size of validation group in Duroc (774) was higher than Yorkshire (471) and Landrace (392–393), which could be an important factor in calculating the genomic prediction accuracy. The validation group size and selection of response variable are important factors on prediction accuracy, which have not been highlighted in the previous genomic prediction studies in pig (Badke et al., 2014; Song et al., 2017, 2019a; Thekkoot et al., 2018; Zhang et al., 2018; Lopez et al., 2019; Aliakbari et al., 2020).

It is evident that ssGBLUP was the most accurate method in five scenarios of Duroc-ADG, Duroc-LMD, Landrace-BFT, Landrace-ADG, and Yorkshire-BFT, and BayesCπ was the most accurate method in three scenarios of Duroc-BFT, Landrace-LMD, and Yorkshire-ADG. However, it should be noted that the accuracies obtained from different genomic prediction methods were not significantly (*P* > 0.05) different in all scenarios. High prediction accuracies obtained from ssGBLUP method in aforementioned scenarios might be due to the blended pedigree and genotypic data used for prediction of GEBVs (Silva et al., 2016; Mrode et al., 2018). The assumption of ssGBLUP method is based on the infinitesimal model of polygenic control of the trait (Karaman et al., 2016, 2018). Although we obtained the higher prediction accuracies for ssGBLUP compared to other methods in five scenarios, our regression coefficients showed considerably underestimated GEBVs for Duroc-BFT (1.60), Duroc-ADG (2.36), and Duroc-LMD (1.48) using ssGBLUP in comparison with the other methods. A reason for these underestimated values in Duroc-ssGBLUP scenarios might be due to the used scaling factors (τ = 1 and ω = 1) to combine *G*^−1^ and $A_{22}^{-1}$ matrices. Alvarenga et al. (2020) indicated that optimization of scaling factors might be helpful for obtaining more unbiased values using ssGBLUP method (Alvarenga et al., 2020). Another possible reason for these underestimated values in Duroc-ssGBLUP scenarios can be due to the preferential treatment to select elite pigs for genotyping in breeding companies. The effect of preferential treatment on level bias was highlighted by former studies in dairy cattle breeding industry (Wiggans et al., 2011; Nordbø et al., 2019). However, it may need more investigation for future studies on swine. In contrast to the GBLUP prediction methods, some genomic prediction methods based on Bayesian approaches such as BayesCπ assume that the genetic variation of a trait is explained by a small number of SNPs (Habier et al., 2007; Hayes et al., 2009b; de los Campos et al., 2013). Therefore, the superiority of BayesCπ in some scenarios might be due to the effective role of SNPs with major effects for Duroc-BFT, Yorkshire-ADG, and Landrace-LMD. Moreover, BayesCπ was the least biased method for five scenarios including Duroc-LMD, Landrace-BFT, Landrace-ADG, Yorkshire-BFT, and Yorkshire-ADG. Based on the superiority of BayesCπ in predictive ability for three scenarios and unbiasedness for five scenarios, it could be concluded that BayesCπ could provide a more dynamic and realistic assumption (Legarra et al., 2011b) for genetic architecture of aforementioned traits, although its long computational time might be a nonpersuasive factor for implementation to the pig industry.

## Conclusion

The accuracies of traditional BLUP, GBLUP, BayesC, ssGBLUP, and BayesCπ methods in a moderate genotyped size of Canadian swine populations were evaluated to compare their predictive abilities. In most scenarios, ssGBLUP and BayesCπ methods demonstrated the highest prediction accuracies and unbiasedness, respectively, although there were no significant differences (*P* > 0.05) among prediction accuracies obtained from these genomic methods in each scenario. These results can be beneficial for implementing the suggested genomic prediction methods for improvement of BFT, LMD, and ADG in swine breeding companies.

## Data Availability Statement

The datasets presented in this article are not readily available because the data are not publicly available due to privacy. Requests to access the datasets should be directed to YM.

## Ethics Statement

Ethical review and approval was not required for the animal study because the animals in this study were raised in commercial farms and only data was provided to this study without any changes to the routine production and thus no animal ethics were needed. Animals were also cared for according to the Canadian Council on Animal Care guidelines.

## Author Contributions

YM, BS, and MJ conceived and designed this experiment. MJ prepared the imputed genotype files. SS wrote the first draft of the manuscript and analyzed the data under supervision of YM, BS, MS, and MJ. SS done visualizations. All authors have read and agreed to the published version of the manuscript.

## Conflict of Interest

MS is employed at Select Sires Inc. This organization did not play any role in the study design, data collection and analysis, decision to publish, or preparation of the manuscript and only provided financial support in the form of MS’s salary. The remaining authors declare that the research was conducted in the absence of any commercial or financial relationships that could be construed as a potential conflict of interest.

## Footnotes

1 https://interbull.org

2 https://www.ccsi.ca/

3 https://slurm.schedmd.com

4 https://docs.scinet.utoronto.ca

## Supplementary Material

Supplementary Figure 1 Heatmap plot of **(A)** scaled genomic and **(B)** pedigree relationship matrices for Duroc group. The means of genomic relationships among reference-validation and validation-validation groups were −0.0047 (range = −0.3065 to 0.6399) and 0.0348 (range = −0.2140 to 0.8164), respectively. The means of pedigree relationships among reference-validation and validation-validation groups were 0.3934 (range = 0.0877 to 0.9339) and 0.4676 (range = 0.4229 to 0.9219), respectively.

Supplementary Figure 2 Heatmap plot of **(A)** scaled genomic and **(B)** pedigree relationship matrices for Landrace group. The means of genomic relationships among reference-validation and validation-validation groups were −0.0037 (range = −0.2064 to 0.6296) and 0.0364 (range = −0.1740 to 0.6711), respectively. The means of pedigree relationships among reference-validation and validation-validation groups were 0.3026 (range = 0.0738 to 0.8396) and 0.3298 (range = 0.1944 to 0.8369), respectively.

Supplementary Figure 3 Heatmap plot of **(A)** scaled genomic and **(B)** pedigree relationship matrices for Yorkshire group. The means of genomic relationships among reference-validation and validation-validation groups were −0.0041 (range = −0.2601 to 0.6921) and 0.0468 (range = −0.1707 to 0.7612), respectively. The means of pedigree relationships among reference-validation and validation-validation groups were 0.2100 (range = 0.0353 to 0.7457) and 0.2553 (range = 0.1160 to 0.7445), respectively.

Supplementary Table 1 The average number of phenotyped boars and gilts per litter for Duroc, Landrace, and Yorkshire groups.

Supplementary Table 2 Estimated variance components (standard errors) to use in best linear unbiased prediction (BLUP), single-step genomic BLUP (ssGBLUP), and genomic BLUP (GBLUP).

Supplementary Table 3 Computational time (h) of genomic BLUP (GBLUP), single-step genomic BLUP (ssGBLUP), BayesC, and BayesCπ methods for back-fat thickness (BFT), average daily gain (ADG), and loin muscle depth (LMD) for Duroc, Landrace, and Yorkshire breeds.

Supplementary Table 4 The results of Tukey tests using Model 5 (*y*_*ij*_ = μ + *M*_*i*_ + *B*_*j*_ + *e*_*ij*_). *y* is the accuracy of prediction for the trait, μ is the overall mean, *M* is the fixed effect of genomic evaluation method, *B* is the fixed effect of breed, and *e* is the random residual effect.
