# Supplementary figures and images for: Genomic Prediction of Average Daily Gain, Back-Fat Thickness, and Loin Muscle Depth Using Different Genomic Tools in Canadian Swine Populations
Source: Front Genet. 2021 Jun 3;12:665344. doi: 10.3389/fgene.2021.665344 (PMC8209496; doi:10.3389/fgene.2021.665344)

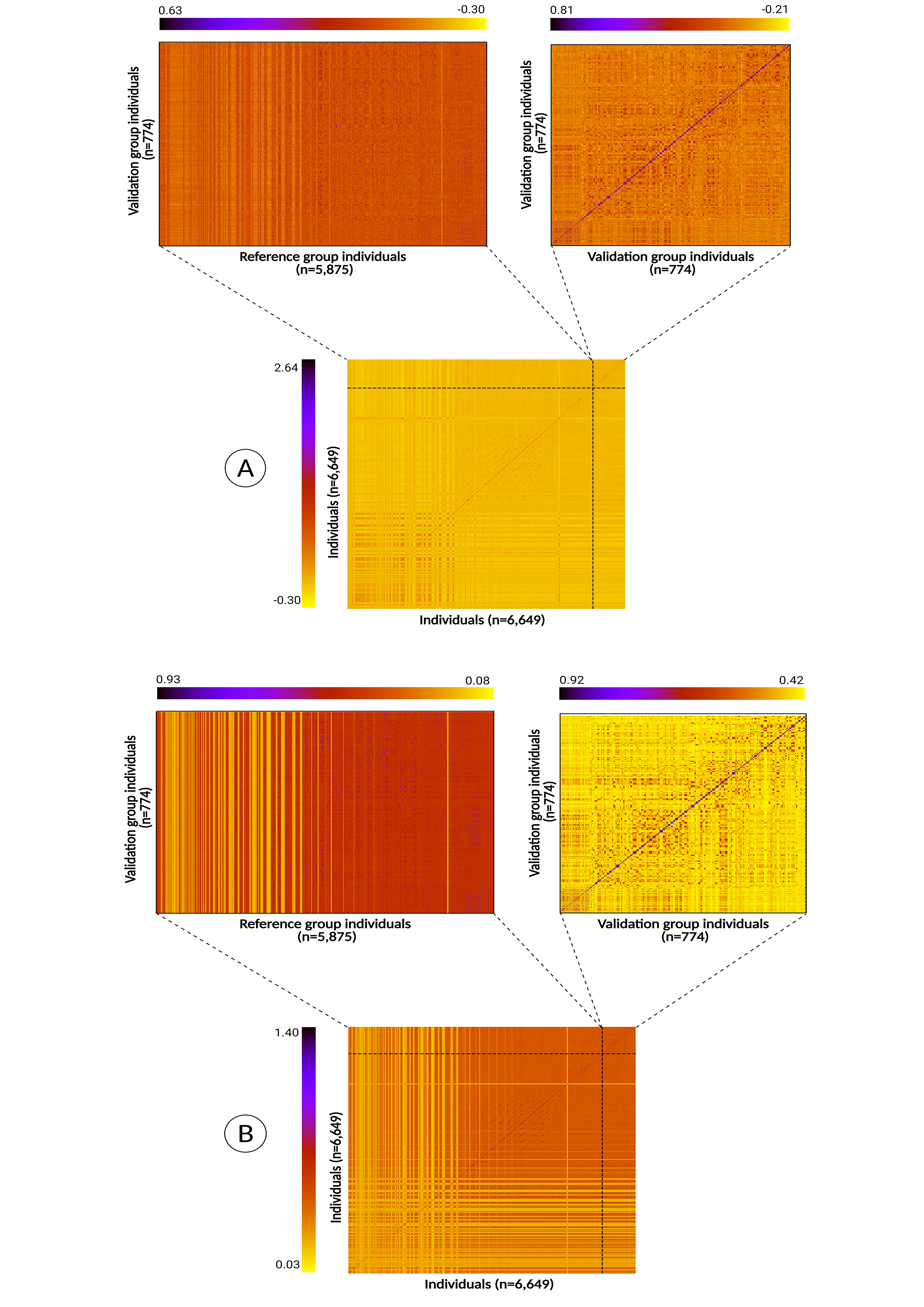

Supplement: Supplementary Figure 1 — Heatmap plot of (A) scaled genomic and (B) pedigree relationship matrices for Duroc group. The means of genomic relationships among reference-validation and validation-validation groups were −0.0047 (range = −0.3065 to 0.6399) and 0.0348 (range = −0.2140 to 0.8164), respectively. The means of pedigree relationships among reference-validation and validation-validation groups were 0.3934 (range = 0.0877 to 0.9339) and 0.4676 (range = 0.4229 to 0.9219), respectively. [file Image_1.JPEG]

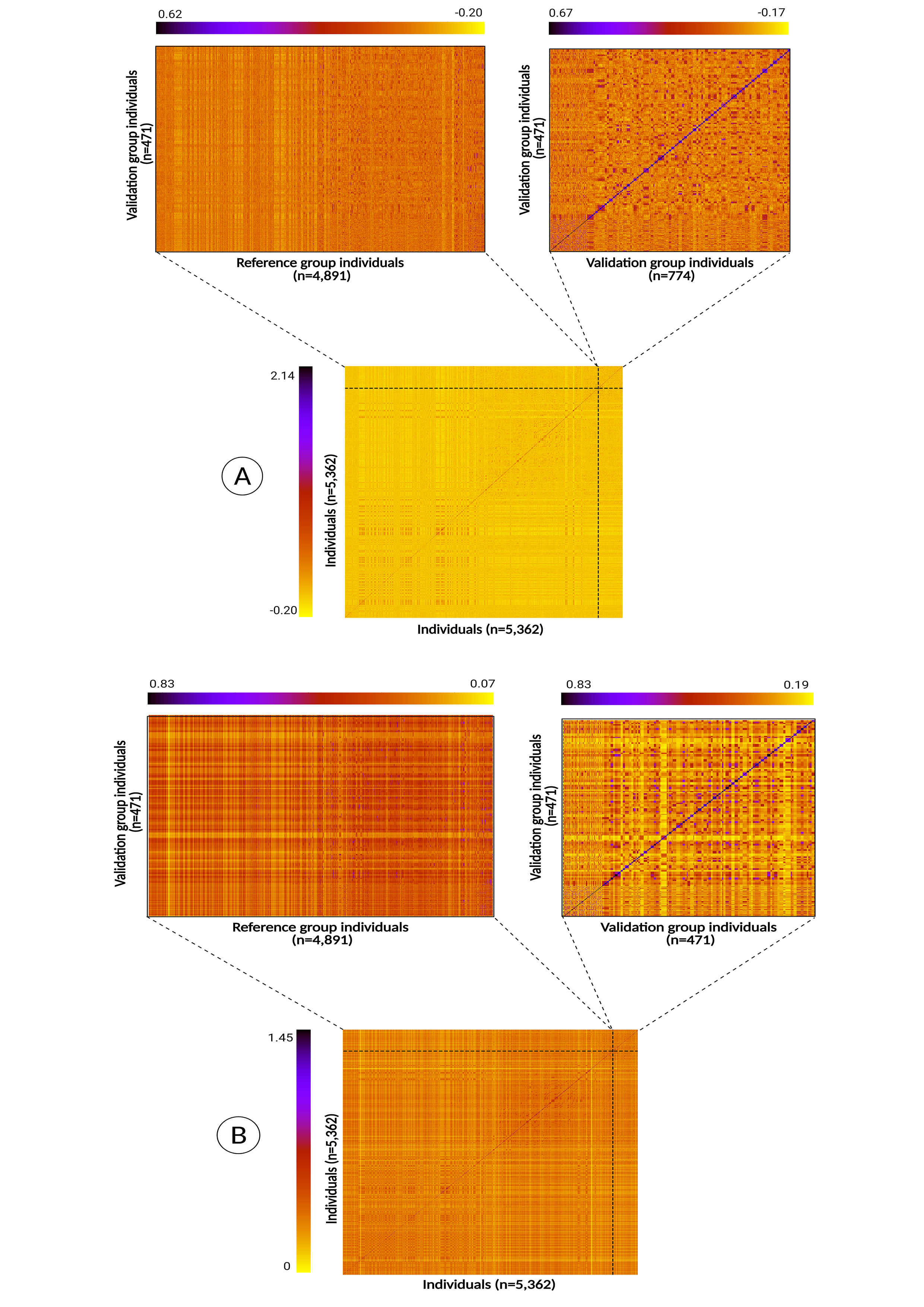

Supplement: Supplementary Figure 2 — Heatmap plot of (A) scaled genomic and (B) pedigree relationship matrices for Landrace group. The means of genomic relationships among reference-validation and validation-validation groups were −0.0037 (range = −0.2064 to 0.6296) and 0.0364 (range = −0.1740 to 0.6711), respectively. The means of pedigree relationships among reference-validation and validation-validation groups were 0.3026 (range = 0.0738 to 0.8396) and 0.3298 (range = 0.1944 to 0.8369), respectively. [file Image_2.JPEG]

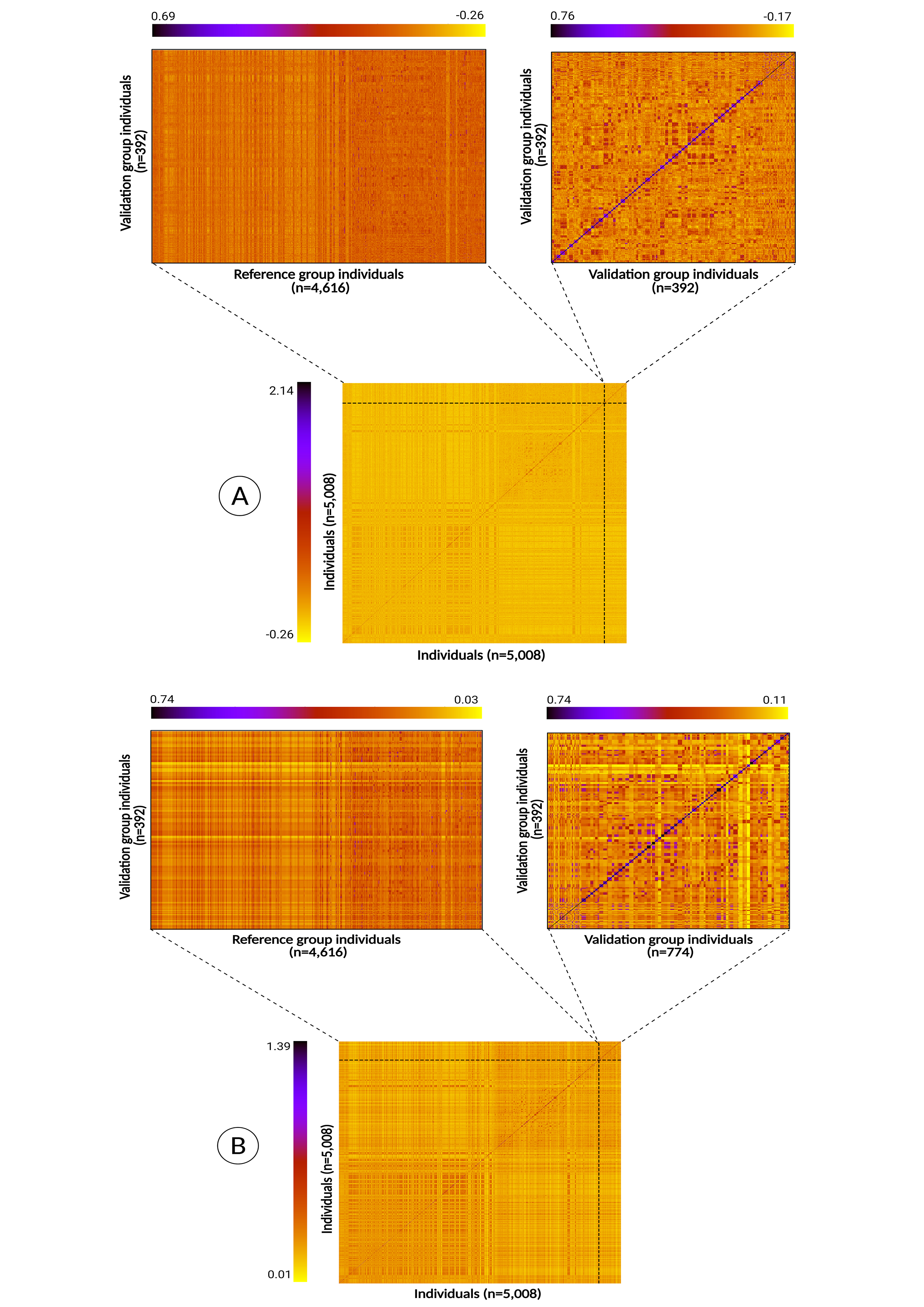

Supplement: Supplementary Figure 3 — Heatmap plot of (A) scaled genomic and (B) pedigree relationship matrices for Yorkshire group. The means of genomic relationships among reference-validation and validation-validation groups were −0.0041 (range = −0.2601 to 0.6921) and 0.0468 (range = −0.1707 to 0.7612), respectively. The means of pedigree relationships among reference-validation and validation-validation groups were 0.2100 (range = 0.0353 to 0.7457) and 0.2553 (range = 0.1160 to 0.7445), respectively. [file Image_3.JPEG]
