# Supplementary material for: Genomic Prediction of Average Daily Gain, Back-Fat Thickness, and Loin Muscle Depth Using Different Genomic Tools in Canadian Swine Populations
Source: Front Genet. 2021 Jun 3;12:665344. doi: 10.3389/fgene.2021.665344 (PMC8209496; doi:10.3389/fgene.2021.665344)
Supplement: Supplementary Table 1 — The average number of phenotyped boars and gilts per litter for Duroc, Landrace, and Yorkshire groups. [file Table_1.docx]

**Supplementary Table 1.** The average number of phenotyped boars and gilts per litter for Duroc, Landrace and Yorkshire breeds.

| **Breed** | **Gender** | **Average number of phenotyped animals per litter** |
| --- | --- | --- |
| **Duroc** | gilt | 3 |
|  | boar | 6 |
| **Landrace** | gilt | 7 |
|  | boar | 3 |
| **Yorkshire** | gilt | 4 |
|  | boar | 2 |
