# Supplementary material for: Genomic Prediction of Average Daily Gain, Back-Fat Thickness, and Loin Muscle Depth Using Different Genomic Tools in Canadian Swine Populations
Source: Front Genet. 2021 Jun 3;12:665344. doi: 10.3389/fgene.2021.665344 (PMC8209496; doi:10.3389/fgene.2021.665344)
Supplement: Supplementary Table 2 — Estimated variance components (standard errors) to use in best linear unbiased prediction (BLUP), single-step genomic BLUP (ssGBLUP), and genomic BLUP (GBLUP). [file Table_2.docx]

**Supplementary Table 2.** Estimated variance components (standard errors) used in best linear unbiased prediction (BULP), genomic BLUP (GBLUP), and single-step genomic BLUP (ssGBLUP).

|  | | **BLUP (before performance test)** | | |  | **BLUP (after performance test)** | | |  | **GBLUP** | |  | **ssGBLUP** | | |
| --- | --- | --- | --- | --- | --- | --- | --- | --- | --- | --- | --- | --- | --- | --- | --- |
| **Trait** | **Breed** | $\boldsymbol{\sigma}_{\boldsymbol{a}}^{\boldsymbol{2}}$ ^1^ | $\boldsymbol{\sigma}_{\boldsymbol{u}}^{\boldsymbol{2}}$ ^2^ | $\boldsymbol{\sigma}_{\boldsymbol{e}}^{\boldsymbol{2}}$ ^3^ |  | $\boldsymbol{\sigma}_{\boldsymbol{a}}^{\boldsymbol{2}}$ | $\boldsymbol{\sigma}_{\boldsymbol{u}}^{\boldsymbol{2}}$ | $\boldsymbol{\sigma}_{\boldsymbol{e}}^{\boldsymbol{2}}$ |  | $\boldsymbol{\sigma}_{\boldsymbol{g}}^{\boldsymbol{2}}$^4^ | $\boldsymbol{\sigma}_{\boldsymbol{e}}^{\boldsymbol{2}}$ |  | $\boldsymbol{\sigma}_{\boldsymbol{g}}^{\boldsymbol{2}}$ | $\boldsymbol{\sigma}_{\boldsymbol{u}}^{\boldsymbol{2}}$ | $\boldsymbol{\sigma}_{\boldsymbol{e}}^{\boldsymbol{2}}$ |
| **BFT** | **Duroc** | 2.12(0.09) | 0.21(0.01) | 1.95(0.04) |  | 2.13(0.08) | 0.21(0.01) | 1.98(0.04) |  | 4.16(0.34) | 8.89(0.19) |  | 2.07(0.07) | 0.22(0.01) | 1.99(0.03) |
|  | **Landrace** | 4.71(0.13) | 0.26(0.01) | 2.43(0.05) |  | 4.78(0.13) | 0.26(0.01) | 2.44(0.05) |  | 2.81(0.21) | 4.14(0.10) |  | 4.54(0.12) | 0.27(0.01) | 2.53(0.05) |
|  | **Yorkshire** | 3.87(0.11) | 0.21(0.01) | 2.64(0.05) |  | 3.96(0.11) | 0.22(0.01) | 2.65(0.05) |  | 3.46(0.26) | 4.49(0.12) |  | 3.91(0.10) | 0.23(0.01) | 2.64(0.04) |
| **ADG** | **Duroc** | 833.64(53.64) | 368.80(14.14) | 1482.20(25.11) |  | 842.04(53.19) | 368.22(13.91) | 1495.20(24.88) |  | 2165.30(261.83) | 11873(247.51) |  | 847.89(48.11) | 381.19(13.94) | 1481.70(21.82) |
|  | **Landrace** | 961.74(45.22) | 333.58(10.02) | 1306.80(20.27) |  | 962.89(44.90) | 338.03(9.98) | 1315.10(20.14) |  | 789.91(74.56) | 2032.90(50.77) |  | 1007.80(43.29) | 336.11( 9.93) | 1292.60(18.91) |
|  | **Yorkshire** | 916.54(42.03) | 410.04(10.67) | 1379.60(19.90) |  | 929.56(41.83) | 412.51(10.57) | 1382.80(19.78) |  | 878.27(96.06) | 2782.60(71.03) |  | 947.21(40.81) | 415.08(10.61) | 1369.80(18.93) |
| **LMD** | **Duroc** | 5.34(0.31) | 1.11(0.07) | 11.76(0.16) |  | 5.37( 0.31) | 1.20(0.07) | 12.13(0.16) |  | 7.57(1.24) | 83.40(1.68) |  | 4.81(0.27) | 1.26(0.07) | 12.04(0.14) |
|  | **Landrace** | 6.48(0.29) | 1.09(0.05) | 12.08(0.14) |  | 6.59(0.29) | 1.10(0.05) | 12.26(0.14) |  | 3.02(0.36) | 14.56(0.34) |  | 6.57(0.29) | 1.15(0.05) | 12.09(0.13) |
|  | **Yorkshire** | 6.64(0.26) | 1.04(0.05) | 11.76(0.13) |  | 6.65(0.26) | 1.08(0.05) | 11.88(0.13) |  | 3.87(0.47) | 16.89(0.41) |  | 6.90(0.27) | 1.07(0.05) | 11.69(0.13) |

1. Variance of additive genetic effects
2. Variance of common litter effects
3. Residual variance
4. Variance of genomic effects
