# Supplementary material for: Genomic Prediction of Average Daily Gain, Back-Fat Thickness, and Loin Muscle Depth Using Different Genomic Tools in Canadian Swine Populations
Source: Front Genet. 2021 Jun 3;12:665344. doi: 10.3389/fgene.2021.665344 (PMC8209496; doi:10.3389/fgene.2021.665344)
Supplement: Supplementary Table 3 — Computational time (h) of genomic BLUP (GBLUP), single-step genomic BLUP (ssGBLUP), BayesC, and BayesCπ methods for back-fat thickness (BFT), average daily gain (ADG), and loin muscle depth (LMD) for Duroc, Landrace, and Yorkshire breeds. [file Table_3.docx]

**Supplementary Table 3.** Computational time (hr) of genomic BLUP (GBLUP), single-step genomic BLUP (ssGBLUP), BayesC and BayesCπ methods for backfat thickness (BFT), average daily gain (ADG) and loin muscle depth (LMD) for Duroc, Landrace and Yorkshire breeds.

| Trait | Breed | GBLUP | ssGBLUP | BayesC | BayesCπ |
| --- | --- | --- | --- | --- | --- |
| BFT | Duroc | 00:00:33 | 00:21:53 | 15:53:11 | 16:20:47 |
|  | Landrace | 00:00:27 | 00:31:43 | 15:15:39 | 16:06:02 |
|  | Yorkshire | 00:00:27 | 00:22:11 | 14:25:25 | 14:16:28 |
| ADG | Duroc | 00:00:36 | 00:19:22 | 16:21:31 | 16:09:34 |
|  | Landrace | 00:00:32 | 00:16:20 | 15:27:03 | 16:21:13 |
|  | Yorkshire | 00:00:30 | 00:16:58 | 14:36:20 | 14:44:02 |
| LMD | Duroc | 00:00:43 | 00:09:54 | 15:53:33 | 16:22:40 |
|  | Landrace | 00:00:37 | 00:16:58 | 15:15:28 | 15:25:15 |
|  | Yorkshire | 00:00:30 | 00:13:45 | 14:21:19 | 15:14:17 |
